# Initial characterization of a transgenic mouse with overexpression of the human D_1_-dopamine receptor in the heart

**DOI:** 10.1007/s00210-023-02901-y

**Published:** 2024-01-04

**Authors:** Lina Maria Rayo Abella, Hannes Jacob, Christin Hesse, Britt Hofmann, Sarah Schneider, Lisa Schindler, Max Keller, Igor B. Buchwalow, CongYu Jin, Pertti Panula, Stefan Dhein, Jan Klimas, Katarína Hadova, Ulrich Gergs, Joachim Neumann

**Affiliations:** 1 Institute for Pharmacology and Toxicology, Medical Faculty, Martin Luther University Halle-Wittenberg, D-06097 Halle (Saale), Germany; 2 Department of Cardiac Surgery, Mid-German Heart Center, University Hospital Halle, D-06097 Halle (Saale), Germany; 3 Institute of Pharmacy, University of Regensburg, D-93053 Regensburg, Germany; 4 Institute for Hematopathology, D-22547 Hamburg, Germany; 5 Scientific and Educational Resource Center for Molecular Morphology, Peoples’ Friendship University of Russia, RU-117198 Moscow, Russia; 6 Department of Anatomy, University of Helsinki, FI-00290 Helsinki, Finland; 7 Rudolf-Boehm Institute for Pharmacology and Toxicology, University Leipzig, D-04107 Leipzig, Germany; 8 Department of Pharmacology and Toxicology, Faculty of Pharmacy, Comenius University, SK-83232 Bratislava, Slovak Republic

**Keywords:** Human D_1_-dopamine receptor, Transgenic mouse heart, Force of contraction

## Abstract

Dopamine can exert effects in the mammalian heart via five different dopamine receptors. There is controversy whether dopamine receptors increase contractility in the human heart. Therefore, we have generated mice that overexpress the human D_1_-dopamine receptor in the heart (D_1_-TG) and hypothesized that dopamine increases force of contraction and beating rate compared to wild-type mice (WT). In D_1_-TG hearts, we ascertained the presence of D_1_-dopamine receptors by autoradiography using [^3^H]SKF 38393. The mRNA for human D_1_-dopamine receptors was present in D_1_-TG hearts and absent in WT. We detected by in-situ-hybridization mRNA for D_1_-dopamine receptors in atrial and ventricular D_1_-TG cardiomyocytes compared to WT but also in human atrial preparations. We noted that in the presence of 10 µM propranolol (to antagonize β-adrenoceptors), dopamine alone and the D_1_- and D_5_-dopamine receptor agonist SKF 38393 (0.1–10 µM cumulatively applied) exerted concentration- and time-dependent positive inotropic effects and positive chronotropic effects in left or right atrial preparations from D_1_-TG. The positive inotropic effects of SKF 38393 in left atrial preparations from D_1_-TG led to an increased rate of relaxation and accompanied by and probably caused by an augmented phosphorylation state of the inhibitory subunit of troponin. In the presence of 0.4 µM propranolol, 1 µM dopamine could increase left ventricular force of contraction in isolated perfused hearts from D_1_-TG. In this model, we have demonstrated a positive inotropic and chronotropic effect of dopamine. Thus, in principle, the human D_1_-dopamine receptor can couple to contractility in the mammalian heart.

## Introduction

**Fig. 1:**
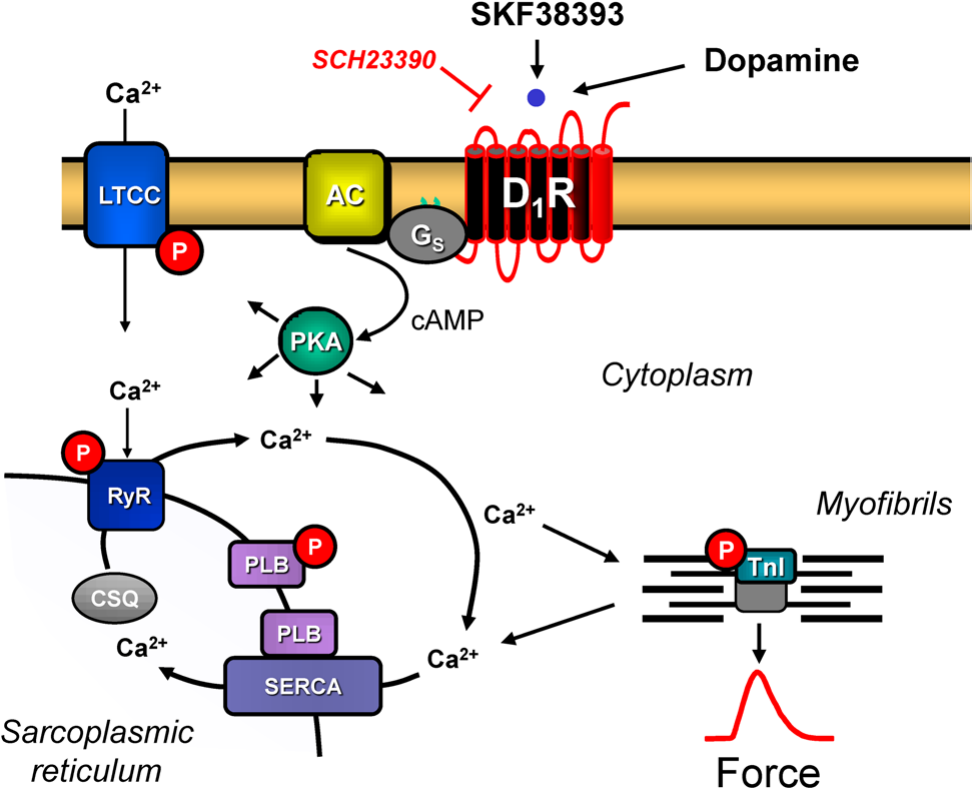
Scheme. D1-dopamine receptors (D1R) are located in the sarcolemma of cardiomyocytes. They can be stimulated by dopamine or the D1R-agonist SKF38393. SCH22390 is an antagonist at D1R. When D1R have been activated, the activity of adenylyl cyclases (AC) is enhanced via GTP-binding stimulatory proteins (Gs). This leads to formation of cAMP and activation of cAMP-dependent protein kinases (PKA). PKA can activate by phosphorylation the L-type calcium ion channels (LTCC) or the ryanodine receptor (RYR) or phospholamban (PLB) or the troponin inhibitor (TnI)

All five currently known dopamine receptor subtypes have been described on RNA-level and/or protein-level in the mammalian heart (review: Neumann et al. 2023a). There is consensus in the literature that D_1_- and D_5_-dopamine receptors can increase the activity of adenylyl cyclases, whereas D_2_-, D_3_-, and D_5_-dopamine receptors can inhibit the activity of adenylyl cyclases (Fig. 1).

Clinically, dopamine, dopamine metabolism, and dopamine receptors are perhaps most relevant in Parkinson’s disease. Moreover, D_1_-dopamine receptors are also targeted by several neuroleptic agents used in psychiatry. D_1_-dopamine receptors at least in the vessel wall are clinically stimulated when dopamine is applied through a perfusor pump in the intensive care unit, usually to increase perfusion of the Arteria renalis in an effort to better the kidney perfusion and thus kidney function (Neumann et al. 2003a). Higher dosages of dopamine in the perfusor can stimulate also adrenergic receptors in the vessel wall, can release stored noradrenaline, and have been used therefore to increase the blood pressure of patients. To summarize, there is a clinical need to understand human cardiac D_1_-dopamine receptors better.

This understanding is complicated by species differences, regional differences, and cellular differences in dopamine receptor function in the heart (review: Neumann et al. 2003a). In the wild-type mouse heart, a direct dopamine receptor-mediated inotropic or chronotropic effect is currently thought to be missing (Yamaguchi et al. 2020).

Besides the D_1_-dopamine receptor (Zhou et al. 1990), we have also to mention in the present context the very similar D_5_-dopamine receptor. The D_5_-receptor was initially named D_1A_-receptor indicating a very close similarity of these two receptors. Currently, it remains functionally impossible to discern D_1_-dopamine receptors and D_5_-dopamine receptors with receptor agonists or antagonists because they are so similar in their ligand affinities (Neumann et al. 2023a): for instance, the D_1_-dopamine receptor agonist SKF 38393 and the D_1_-dopamine receptor antagonist SCH23390 exhibit practically identical affinities for D_1_-dopamine and D_5_-dopamine receptors in ligand binding studies (Neumann et al. 2023a). Hence, with pharmacological tools, it is currently impossible to decide whether a functional response in the heart to dopamine is D_1_-dopamine receptor- or D_5_-dopamine receptor-mediated.

In humans, D_1_-dopamine receptors were detected in cardiac atrium and ventricle on mRNA and protein levels in Western blots but also using in situ hybridization and immunohistochemistry (Ozono et al. 1997). More recently, using single-cell RT-PCR, one has detected D_1_-dopamine receptors in single cardiomyocytes from the human ventricle (Yamaguchi et al. 2020). Interestingly, the authors report that the mRNA level of the D_1_-dopamine receptors increased with the deterioration of the ventricular function in patients (by studying surgical samples, Yamaguchi et al. 2020). Their data present thus strong evidence for a pathophysiological relevance of D_1_-dopamine receptors in the human heart.

However, it appears that D_1_-dopamine receptors are unable to increase force of contraction in the human heart. For instance, in human ventricular tissue, any positive inotropic effect of dopamine was explained by dopamine acting on β_1_- and β_2_-adrenoceptors (Brown et al. 1985; Port et al. 1990; Bravo et al. 1991). In the human atrium, dopamine like in the ventricle stimulated β_1_- and β_2_-adrenoceptors. In addition, dopamine was also an agonist at α_1_-adrenoceptors in human right atrial muscle preparations (Wagner et al. 1980; Deighton et al. 1992; Bravo et al. 1991). Moreover, in human atrium as well as in human ventricle, dopamine can release noradrenaline and thereby stimulated adrenergic receptors. Thus, dopamine is also an indirect sympathomimetic agent in the human heart (Rump et al 1995; Brown et al. 1985; Port et al. 1990).

The situation in animal hearts is similar. Dopamine receptors have been identified in animal hearts: D_1_-dopamine receptors were immunologically detected on cardiomyocytes in the rat (Ozono et al. 1996) or as mRNA in homogenates from mouse hearts (Yamaguchi et al. 2020). However, in the heart of the guinea pig, rat, dog, pig, and rabbit, dopamine acts via β-adrenoceptors and not via dopamine-receptors to raise force of contraction and beating rate. At least in rabbits, dopamine also acts an agonist at cardiac α-adrenoceptors (review: Neumann et al. 2023a).

In order to obtain an animal model to study the human D_1_-dopamine receptor in its contractile function, mice with inducible overexpression of D_1_-dopamine receptor in the heart have been recently generated by others (Yamaguchi et al. 2020). These authors activated the transgene (the D_1_-dopamine receptor in the heart) with a “tet-off” system. In other words, they usually fed mice with tetracycline containing water. When the mice were about 1 month of age and older, they withheld tetracycline in the feeding water and then the D_1_-dopamine-receptor was induced in the heart of transgenic animals. They presented cumulative evidence that D_1_-dopamine receptor stimulation led to arrhythmias in their transgenic mice compared to appropriate controls (Yamaguchi et al. 2020). However, they did not study in their model the effect of dopamine on contractility in the isolated heart or in isolated atrial preparations and whether these effects were D_1_-dopamine receptor mediated.

Therefore, we generated transgenic mice and describe them here for the first time, that constitutionally overexpress the D_1_-dopamine receptors in the heart (D_1_-TG). Hence, we tested in this study the simply the hypothesis that dopamine would increase force of contraction and beating rate in D_1_-TG. This would prove that in principle the human D_1_-receptor can increase cardiac contractility, irrespective of the action of dopamine on other cardiac receptors. A progress report of this work has been published in abstract form (Rayo Abella et al. 2023).

## Materials and methods

### Generation of transgenic mice

The investigation conforms to the Guide for the Care and Use of Laboratory Animals published by the National Research Council (2011). Animals were maintained and handled according to approved protocols of the animal welfare committees of the University of Halle-Wittenberg, Germany. The generation and initial characterization of the transgenic mice was similar as with other transgenes described before (Gergs et al. 2010, 2019b). In brief, the human D_1_-dopamine receptor cDNA (Gene bank number NM_000794.5) was inserted into a mouse cardiac α-myosin heavy chain long promoter expression cassette including a rabbit β-globin polyadenylation signal. The linearized and purified construct was injected into the pronuclei of single-cell fertilized CD1 mouse embryos. From several D_1_-transgenic founder animals, one line was established and further characterized in the present study. For all experiments, adult transgenic mice and WT littermates of both sexes were used.

### Contractile studies in mice

Spontaneously beating right atrial preparations from mice were used to study any chronotropic effects. Left atrial preparations from mice were used to study inotropic effects. The right or left atrial preparations from the mice were isolated and moved into organ baths as previously described (Gergs et al. 2013; Neumann et al. 2003). In brief, murine right or left atrial preparations were dissected from the isolated whole heart, mounted vertically under isometric conditions in organ baths of 10 ml volume that were jacketed with water heated such that the organ bath temperature was 37 °C. The right atrial preparations were allowed to beat spontaneously. The left atrial preparations were electrically stimulated with a voltage about 10% above threshold, and the length of the stimulation impulse was 5 ms. Developed tension was measured by using a bridge amplifier, digitized on a personal computer and beating rates and other contractile parameters were plotted and analyzed with a commercial software (Labchart from AD instruments, purchased in Germany). The bathing solution of the organ baths contained 119.8 mM NaCI, 5.4 mM KCI, 1.8 mM CaCl_2_, 1.05 mM MgCl_2_, 0.42 mM NaH_2_PO_4_, 22.6 mM NaHCO_3_, 0.05 mM Na_2_EDTA, 0.28 mM ascorbic acid, and 5.05 mM glucose. The solution was continuously gassed with 95% O_2_ and 5% CO_2_ and maintained at 37 °C and pH 7.4 (Neumann et al. 1998, 2003; Kirchhefer et al. 2004).

The drug application was as follows. After equilibration was reached, in some experiments (as delineated in the appropriate legends), propranolol was applied. After 10 min, we cumulatively added to left atrial or right atrial preparations dopamine or SKF 38393 or dopamine to establish concentration–response curves. In some experiments, these effects were attenuated by applying the antagonist SCH 23390. At the maximum of the inotropic response to the agonists, atrial preparations were quickly brought to the temperature of liquid nitrogen and stored for future analysis at – 80 °C (Fig. 3). Usually, the atria were used in contraction studies and the corresponding ventricles were routinely rapidly frozen in liquid nitrogen at the start of the contraction experiments (for mRNA isolation) or put in the solvent required for embedding and histology (see below).

### Langendorff perfusion

As described by our group (Dörner et al. 2021, Gergs et al. 2004, 2010, 2019a), isolated whole mouse hearts were retrogradely perfused with the same buffer as in “Contractile studies in mice” section above. Hearts were allowed to beat by themselves. Force was monitored from the apex cordis by a hook connected to an electronic force monitor, digitized and analyzed (Labchart from AD Instruments, Germany). Perfusion of hearts with drugs took place with a syringe connected to a pump. This pump was connected as a bypass with the aorta. At the end of experiments, hearts were freeze-clamped in liquid nitrogen to stop any phosphorylation reactions. Frozen samples were kept at – 80 °C until biochemical analysis.

### Human samples

The study in patients complies with the Declaration of Helsinki and has been approved by the local ethics committee (hm-bü 04.08.2005). Informed consent was obtained from all patients included in the study. For the same studies as for mouse atrial preparations, human atrial preparations were used from a male patient (66 years old) who underwent bypass surgery due to coronary heart disease. The tissue was rapidly transferred to the laboratory for embedding and used for in situ hybridization.

### Western blotting

The homogenization of the samples, protein measurements, electrophoresis, primary and secondary antibody incubation, and quantification were performed following our previously established protocols (Gergs et al. 2009, 2019a,b; Boknik et al. 2018). First antibodies were rabbit polyclonal anti-calsequestrin (CSQ) antibody, #ab3516, Abcam, Cambridge, UK (diluted 1:20000), rabbit polyclonal anti-DRD1, #bs-1007R, Bioss, Woburn, MA, USA (dilution 1:500), and rabbit polyclonal anti-phospho-troponin I (P-TnI) antibody, #4004, Cell Signaling Technology Europe, Leiden, The Netherlands (diluted 1:5000).

As controls in some Western blotting experiments, cardiac homogenates from D_1_ receptor knock-out mice were used. Cardiac samples of D_1_ knock-out mice were kindly provided by Jean-Antoine Girault, Insern Research Director; Institute du Fer à Moulin, Paris, France.

### Autoradiography

Mouse left and right atria and ventricle from D_1_-TG and WT mice were inserted into Tissue-Tek (Sakura Europe, Alphen aan den Rijn, The Netherlands) and frozen on cork plates, and stored at – 20 °C or – 78 °C. Cryosections (12 μm) were obtained at – 14 °C with a 2800 Frigocut E freezing microtome (Leica Biosystems, Nussloch, Germany). Three or four adjacent sections were mounted on one microscopic slide (SuperfrostPlus Adhesion Microscope Slides, 75 × 25 × 1 mm; Epredia, Breda, The Netherlands); the slides were kept 1–2 min in a chamber of 100% humidity (quadriPERM culture dishes; Sarstedt, Nümbrecht, Germany) and then carefully covered with a tris(hydroxymethyl)aminomethane (Tris) buffer (50 mM Tris, 120 mM NaCl, 5 mM KCl, 2 mM CaCl_2_, and 1 mM MgCl_2_, pH 7.4) supplemented with 1% bovine serum albumin (Serva, Heidelberg, Germany) (in the following referred to as binding buffer). After 5–15 min, the binding buffer was carefully removed (slides were put uprightly lengthwise on a paper towel for ca. 1 min), the slides were placed back in a chamber of 100% humidity, and the sections were carefully covered with binding buffer containing the selective D_1_-dopamine receptor antagonist [^3^H]SCH23390 (Novandi, Södertälje, Sweden; specific activity: 2.97 TBq/mmol, reported *K*_d_ value (hD_1_R): 630 pM) (Zhou et al.^1990^) (5 nM) to determine total binding, or with binding buffer containing [^3^H]SCH23390 (5 nM) and the D_1_ dopamine receptor antagonist butaclamol (10 µM) to determine unspecific binding. The sections were incubated in a humidity chamber at room temperature for 60 min (during incubation, the chamber was slightly tilted every 5 min to carefully move the binding buffer on the sections). After incubation, the largest part of the binding buffer was drained, the slides were immersed three times into ice-cold PBS (three separate vessels, each 10 s) followed by immersion in ice-cold distilled water for 10 s. The slides were put uprightly lengthwise on a paper towel for 10 min, remaining liquid at the edge of the slides was removed, and the slides were kept in a desiccator over P_4_O_10_ for at least 24 h. The slides were set in close contact with a TR 2025 E Cytiva BAS storage phosphor screen (20 × 25 cm, Fisher Scientific, Schwerte, Germany) using an X-ray film cassette and stored in the dark for 21 days. The autoradiographic image was generated from the screen using a Typhoon FLA 9500 biomolecular imager (GE Healthcare Life Sciences) at a resolution of 25 µm and a photomultiplier voltage of 1000 V.

### PCR

Frozen ventricles from D_1_-TG and WT mice were pulverized mechanically in liquid nitrogen and were processed by acid phenol-guanidinium thiocyanate-chloroform extraction (TRI Reagent^®^, Sigma-Aldrich, St. Louis, MO, USA) according to the manufacturer’s instructions. RNA was precipitated from aqueous phase by isopropanol and washed twice with ethanol. For RNA quality control, samples were tested by electrophoresis in 2% agarose gel (Agarose, Sigma-Aldrich, USA). Intact RNA samples were reverse-transcribed using high capacity cDNA Reverse Transcription Kit with RNAse inhibitors (Applied Biosystems, Grand Island, NY, USA). Quantitative real-time PCR (RT-qPCR) analysis was performed using StepOnePlus™ Real-Time PCR System (Thermo Fisher Scientific, USA) with SYBR™ Select Master Mix (Thermo Fisher Scientific, USA). Expression of exogenous and endogenous dopamine receptor 1 (human DRD1 versus mouse Drd1 respectively) in the ventricles was examined using gene-specific primers (Forward: TACAGACTTTGCCCTGCGAC, Reverse: CTTGGAGATGGAGCCTCGTG; and Forward: AAGTGACTCTAAAGCAAGGGCA, Reverse: AGTCACTTTTCGGGGATGCT, respectively). The primers were designed using Primer-BLAST (PMID: 22708584).

### In situ hybridization

A 591 bp-long probe complimentary to 138–728 bp of the coding region of human D_1_-dopamine receptor were PCR-amplified from human cDNA 1st-strand, and a 773 bp-long probe complimentary to 565–1337 bp of the coding region of mouse D_1_-dopamine receptor were PCR-amplified from mouse cDNA 1st-strand. Both human and mouse probes were cloned into the pGEMT-easy vector between T7 and SP6 promoters. Plasmid clones was linearized at either ends of the insertion and served as templates for in vitro transcription of DIG-labeled ribo-probes by T7 RNA polymerase (produced DIG-labeled antisense *D1 ribo*-probes) or SP6 RNA polymerase (produced DIG-labeled sense *D1 ribo*-probes used as controls).

Hearts from D_1_-TG and wild-type mice, and human atrial muscle strips were fixed with 4% PFA, infiltrated with paraffin and embedded into paraffin wax blocks. Then, 5-µm-thick paraffin sections were deparaffinized, rehydrated, and treated sequentially with 4% PFA and proteinase K (10 µg/ml), then hybridized overnight at 52 °C in a reaction mixture of the labeled probe (1 µg/ml) and hybridization solution.

Paraffin sections were deparaffinized, rehydrated, and treated sequentially with 4% PFA, proteinase K (10 µg/ml) and 4% PFA, then dehydrated again. Dried sections were hybridized overnight at 52 °C in a reaction mixture of the labeled probe (1 µg/ml) and hybridization solution. After hybridization, sections were washed with 1 × SSC at 70 °C, cooled down to RT, pre-incubated with blocking solution, then incubated overnight at 4 °C in blocking solution with anti-DIG antiserum-Ab Fab fragment (1:5000 dilution). After incubation, sections were washed with PBS-T, pre-incubated in staining buffer, and stained by NBT (0.23 mg/ml) and BCIP (0.175 mg/ml) mixture in staining buffer. Sections were then washed and coverslipped with mounting medium (Dako, S3023) for microscopy.

### Histology

Cardiac samples from D_1_-TG and WT mice were fixed in buffered 4% formaldehyde and embedded in paraffin. Thereafter, 2-µm-thick paraffin tissue sections were prepared, deparaffinized with xylene, and graded ethanols and for histological analysis, tissue sections were stained with hematoxylin-eosin (HE) and to detect fibrosis with Masson-Goldner trichrome stain (MG). For all washing steps and dilutions, phosphate-buffered saline was used. For visualization and image processing, a Zeiss microscope “Axio Imager Z1” equipped with an AxioCam digital microscope camera and the AxioVision image processing software were used (Carl Zeiss Vision, Germany). Images shown are representative of 3 independent experiments which gave similar results.

### Data analysis

Data shown are means ± standard deviation (SD). Statistical significance was estimated using the analysis of variance followed by Bonferroni’s *t* test as appropriate. A *p* value < 0.05 was considered to be significant.

### Drugs and materials

The drugs (±)-isoprenaline tartrate, dopamine hydrochloride, (±)-SKF 38393 hydrochloride, and R(+)-SCH23390 hydrochloride were purchased from Sigma-Aldrich (Germany). All other chemicals were of the highest purity grade commercially available. Deionized water was used throughout the experiments. Stock solutions were prepared fresh daily.

## Results

### Autoradiography

**Fig. 2:**
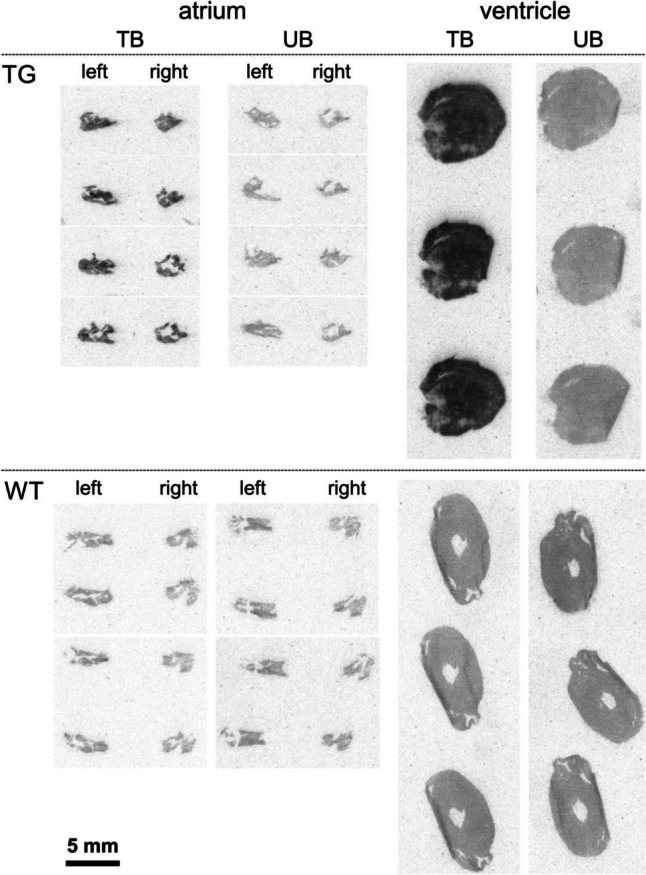

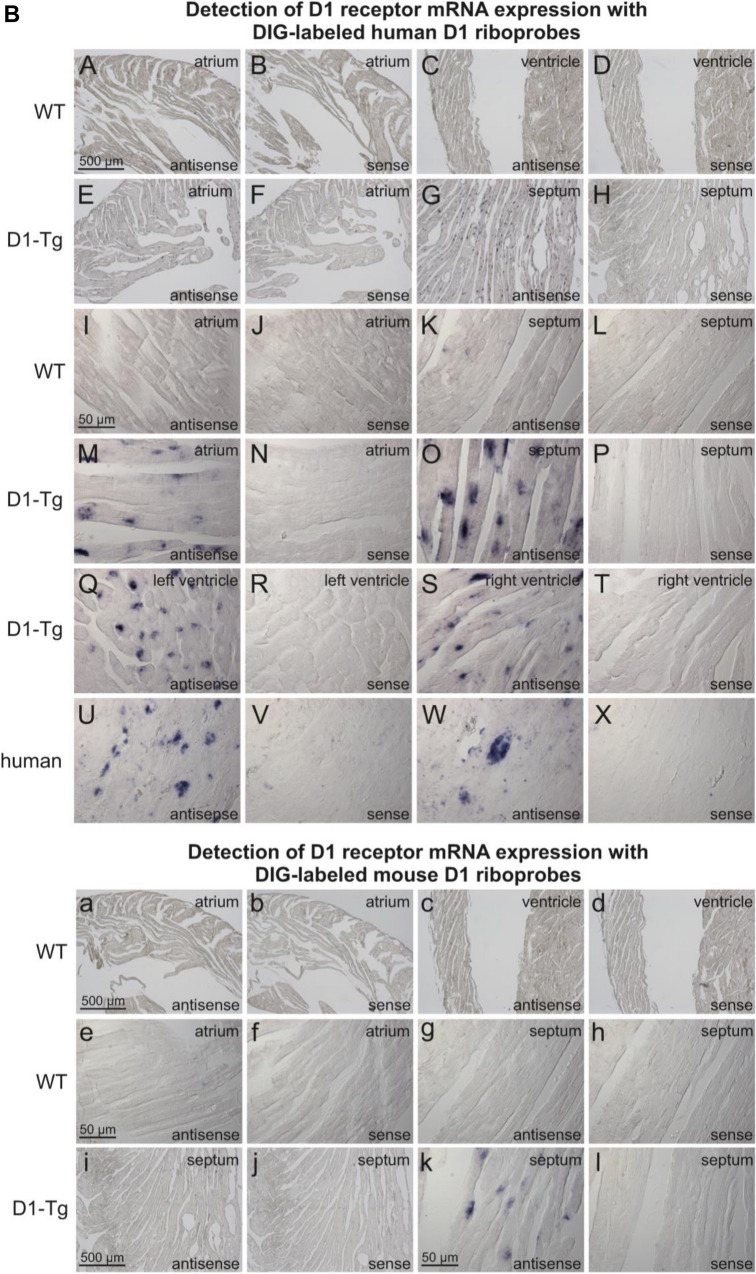

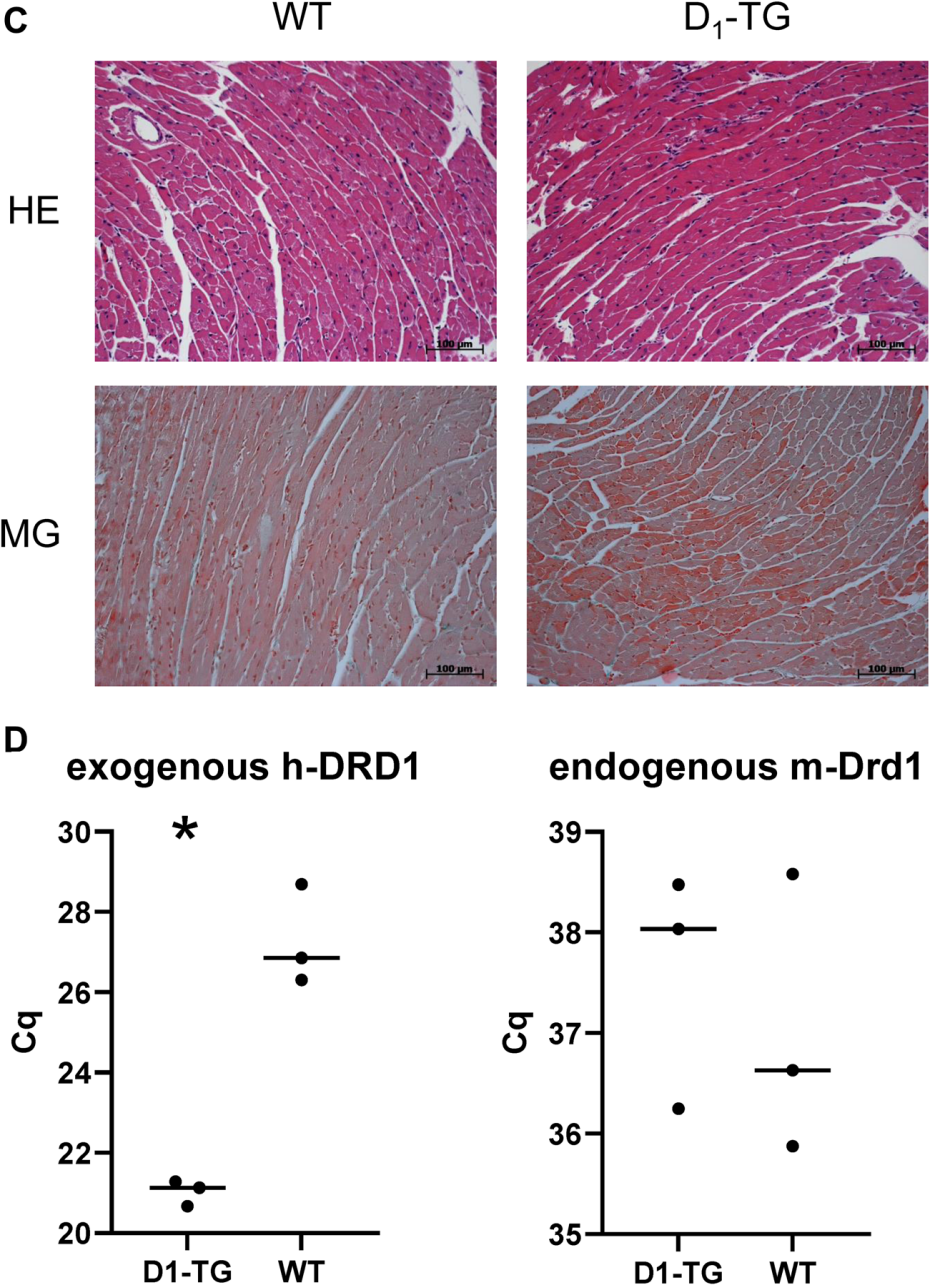
**A** Autoradiography. Representative images of autoradiography of D_1_-TG and WT mouse heart sections labeled with 5 nM [^3^H]SCH23390 (total binding, TB) or 5 nM [^3^H]SCH23390 + 10 M butaclamol (unspecific binding, UB). **B** In situ hybridization. Expression of D_1_-dopamine receptor mRNA detected by DIG-labelled human D_1_-dopamine receptor antisense probes (A-X) and DIG-labelled mouse D_1_-dopamine receptor antisense probes (a-l). DIG-labeled sense probes were used as controls. (A-H): Low magnification microscopic images, scale bar = 500 µm. mRNA expression of D_1_-dopamine receptor was not detected in WT mouse atria (A) and ventricles (C); whereas expression of human D_1_-dopamine receptor mRNA was detected in D_1_-Tg mouse atria (E) and ventricles including interventricular septum (G). (B, D, F, H) are corresponding control sections hybridized with DIG-labeled sense probes. (I-X): High magnification microscopic images, scale bar = 50 µm. No or very scarce and weak D_1_-dopamine mRNA expression was detected in WT mouse atria (I) and interventricular septum (K), while abundant human D_1_-dopamine mRNA expression was detected in atriums (M), interventricular septum (O), and ventricle walls (Q, S). (J, L, N, P, R, T) are corresponding control sections hybridized with DIG-labeled sense probes. D_1_-dopamine mRNA expression was also detected in human cardiomyocytes (U, W); while no signal was seen in corresponding control sections hybridized with DIG-labeled sense probes (V, X). (a-d): Low magnification microscopic images, scale bar = 500 µm. (e-h): High magnification microscopic images, scale bar = 50 µm. Expression of mouse D_1_-dopamine receptor mRNA was not detected in WT mouse atria (a, e) and ventricles (c, g); (b, d, f, h) are corresponding control sections hybridized with DIG-labeled sense probes. Low magnification images (i and j, scale bar = 500 µm) and high magnification images (k and l, scale bar = 50 µm) showed that the mouse antisense probe also detected human D_1_-dopamine mRNA expression in D_1_-TG mouse interventricular septum (i, k), while the mouse sense probe (control) did not detect any specific signal (j, l). **C**: Histology. Histological staining of myocardial sections with hematoxylin-eosin (HE) or Masson-Goldner trichome (MG) staining revealed no apparent abnormalities or signs of fibrosis, neither in WT nor in D_1_-TG. Scale bar = 100 µm. **D**: PCR. Scatter plots with means for the expression of exogenous transgenic D_1_-dopamine receptors (human-DRD1, left hand side) or endogenous mouse D_1_-dopamine receptors. Ordinates indicate the amount of the mRNA as inferred from the amplification cycles (Cq). *indicates significant differences between the mRNA from the whole heart of D_1_-TG versus WT

Autoradiography studies, using the D_1_/D_5_ receptor radioligand [^3^H]SCH23390, resulted in a clear difference between total and unspecific binding for D_1_-TG, indicating D_1_ receptor expression (Fig. 2A). In the case of WT, total and unspecific binding were not distinguishable, demonstrating the absence or very low levels of D_1_ (and D_5_) receptors (Fig. 2A).

### In situ hybridization

The mRNA expression of human D_1_-dopamine receptor was detected in cardiomyocytes of D_1_-TG mice by DIG-labelled human D_1_-antisense ribo-probes (Fig. 2B: E-H, M-T), while no or very scarce signal was detected in cardiomyocytes of WT mice. Moreover, these same probes also detected D_1_-dopamine receptor mRNA expression in human cardiomyocytes (Fig. 2B: U-X). No specific signal was detected in control sections hybridized with DIG-labeled sense probes.

Hybridization with DIG-labelled mouse D_1_-dopamine antisense ribo-probes detected no or very scarce mRNA expression of mouse D_1_-dopamine receptor in WT mice (Fig. 2B: a-h), and no specific signal was detected in control sections hybridized with DIG-labeled sense probes. This observation indicates that there is no or very low level expression of endogenous D_1_-dopamine receptor in mouse heart. On the other hand, some D_1_-dopamine mRNA expression was detected by these mouse antisense probes in D_1_-Tg mice, though the intensity of expression was lower than that detected by the human D_1_ antisense probes (Fig. 2B: i-l). Since the sequence of the mouse D_1_ antisense probe is 86.5% identical to the reverse complimentary sequence of a 773 bp region of human D_1_ receptor mRNA (565–1337 bp of the coding region), this probe is also able to hybridize to 565–1337 bp of the coding region of human D_1_-dopamine receptor mRNA and detect its mRNA expression, but with lower efficiency. Please note that we detected D_1_-dopamine receptors with this method in human atrium (inset U and W) probably in cardiomyocytes.

### Histology

Histological staining was performed for three D_1_-TG and WT mouse heart samples each. Staining of the myocardial sections with HE or Masson-Goldner trichome staining revealed no apparent abnormalities or signs of fibrosis, neither in WT nor in D_1_-TG. There was no difference between D_1_-TG and WT samples (Fig. 2C).

### PCR

The expression of the endogenous mouse D_1_-dopamine receptor was similar in cardiac homogenates from D_1_-TG and WT. There was a high signal of mRNA for the exogenous, human D_1_-dopamine receptor in D_1_-TG but only a very small, probably unspecific signal in for exogenous, human D_1_-dopamine receptor in WT (Fig. 2D). The expression of the endogenous mouse D_1_-dopamine receptor was not different in WT and D_1_-TG, suggesting the endogenous receptor is not downregulated in a compensatory fashion in D_1_-TG. Moreover, it is apparent that in D_1_-TG, much more mRNA for the transgenic D_1_-dopamine receptor than for the endogenous receptor is present. However, because we detected also endogenous D_1_-dopamine receptors in WT (even though the expression was very low), this is consistent with our contractile data (see below) where we also detect D_1_-dopamine receptors at least to a very minor functional extent in the atrium of WT.

### Western blots

**Fig. 3:**
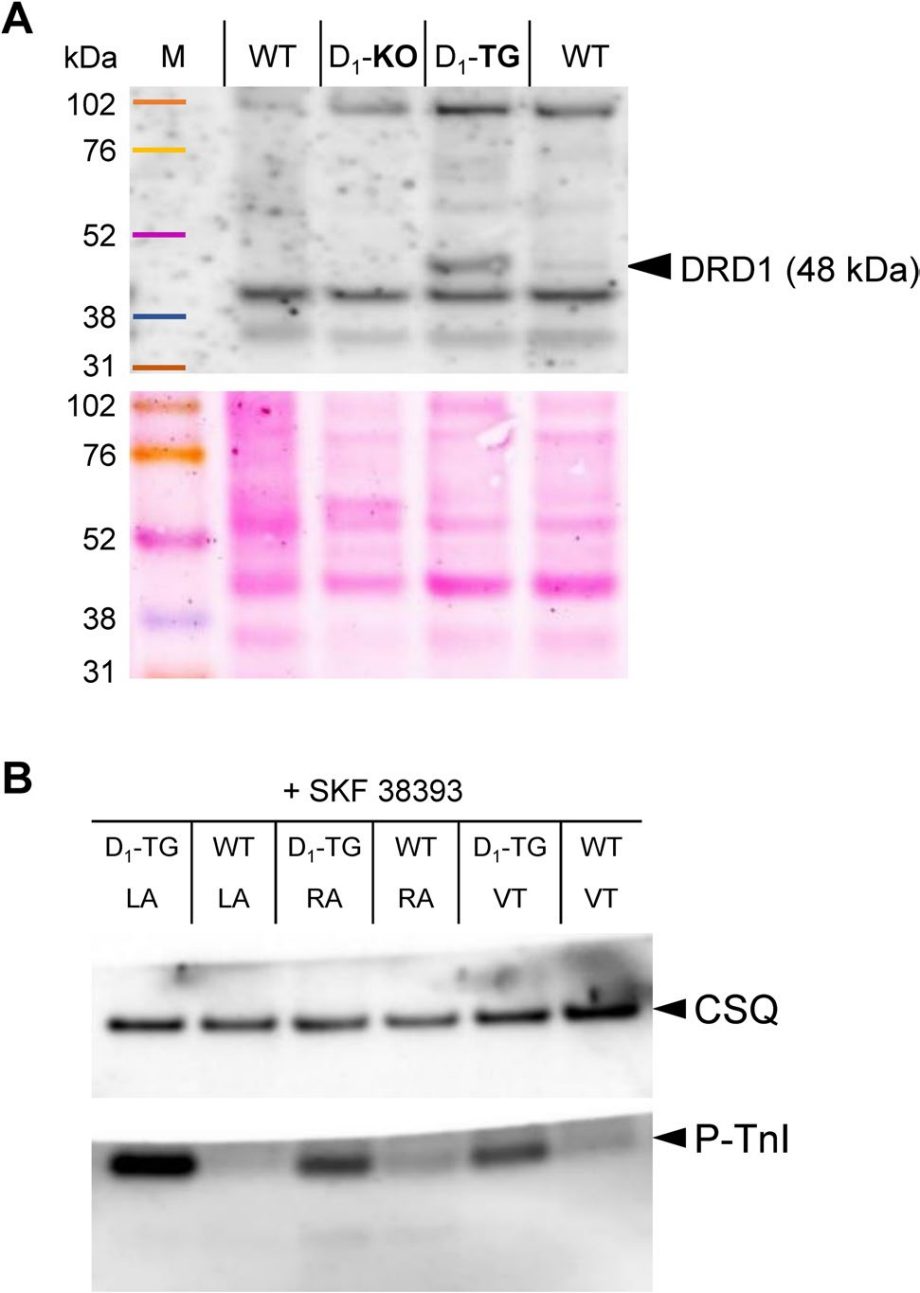
Western blots. **A** A typical Western blot for the D_1_ receptor in hearts from WT, D_1_-TG, and D_1_-KO after incubation with the D_1_-dopamine receptor antibody is seen. The corresponding band for the D_1_ receptor (DRD1) is labelled with an arrow. As loading control, the Ponceau S-stained membrane is shown below. **B** Typical Western blots for regulatory proteins in hearts from WT and D_1_-TG after incubation with the D_1_-dopamine receptor agonist SKF 38393 are seen. Western blots depict the phosphorylation state of inhibitory subunit of troponin (P-TnI) with arrows. As a loading control, we assessed the protein expression of cardiac calsequestrin (CSQ) by cutting horizontally the lanes of the blot and incubating the lower and upper halves with different primary antibodies. *M* rainbow marker, *LA* left atrium, *RA* right atrium, *VT* ventricle

In Fig. 3A, a Western blot is shown that detects the D_1_ receptor in a D_1_-TG heart. A fine band at the corresponding height can be seen in one WT sample. As negative control, a cardiac sample of a D_1_ knockout (D_1_-KO) mouse was used, where no signal was detectable at the corresponding height (Fig. 3A). The expression of SR proteins was similar in corresponding cardiac regions. That is, the expressional levels of phospholamban, inhibitory subunit of troponin (TnI), sarcoplasmic reticulum ATPase (SERCA), and calsequestrin (CSQ) were similar in Western blots from WT and D_1_-TG (data not shown). However, the phosphorylation on serine 23/24 of the inhibitory subunit of troponin (P-TnI) in left and right atrial as well as ventricular preparations was stimulated by SKF 38399 in contracting preparations of D_1_-TG compared to WT (Fig. 3B).

### Contraction in left atrium of D_1_-TG

**Fig. 4:**
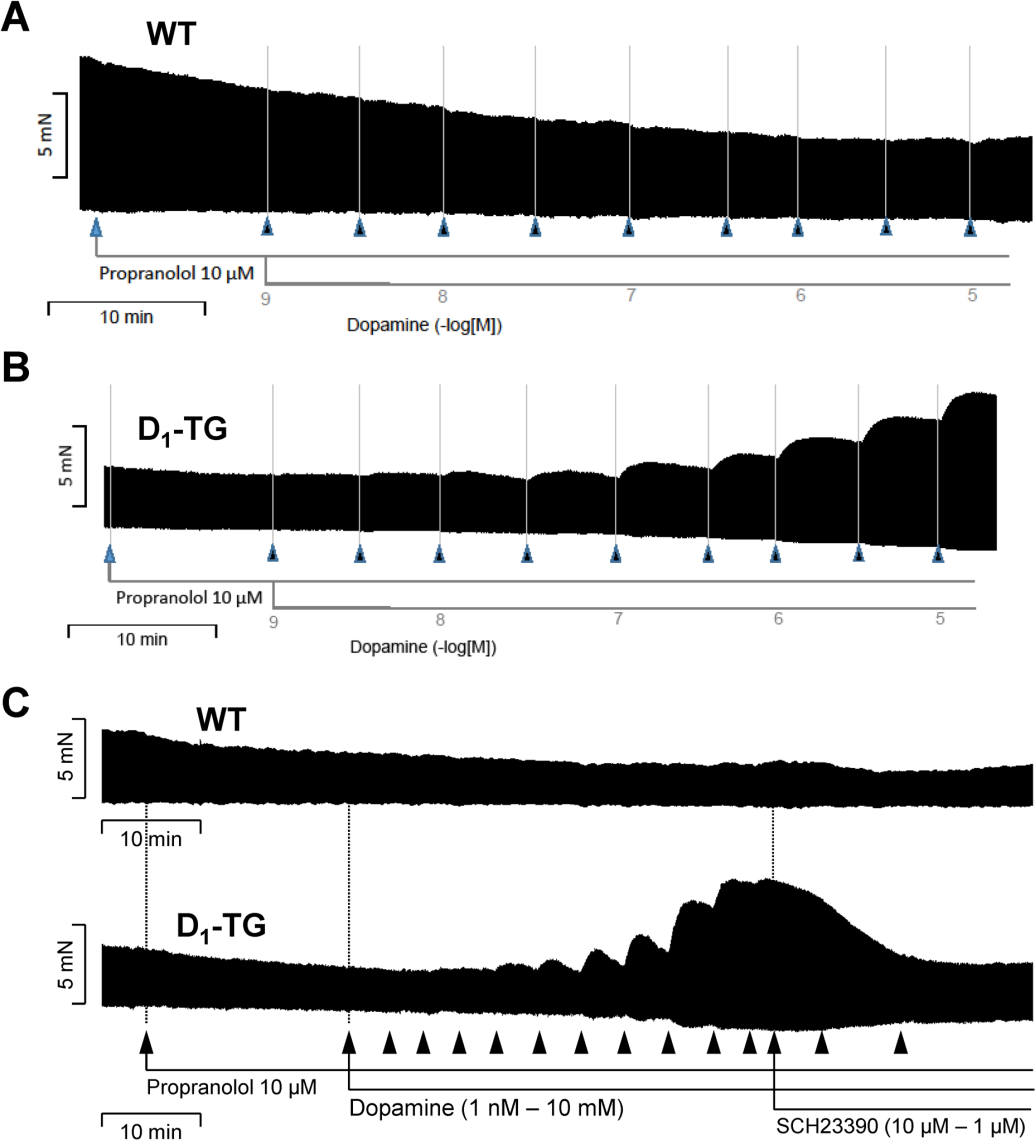
Original recordings: contractile effects of dopamine. Isolated electrically stimulated left atrial preparations from WT (**A**) and D_1_-TG (**B**) were studied. Ordinates give force of contraction in millinewton (mN). Horizontal bar indicates time in minutes (min). First propranolol alone was given (10 µM). After 10 min of pre-treatment dopamine was cumulatively applied. Time points of addition of dopamine are indicated with arrows pointed upwards. Concentrations of dopamine are indicated as negative decadic logarithms. Dopamine increases force of contraction only in preparations from D_1_-TG. **C** The same experiments were carried out here as in (**A**) and (**B**), but finally a concentration-response-curve with the D_1_ receptor antagonist SCH23390 was added

**Fig. 5:**
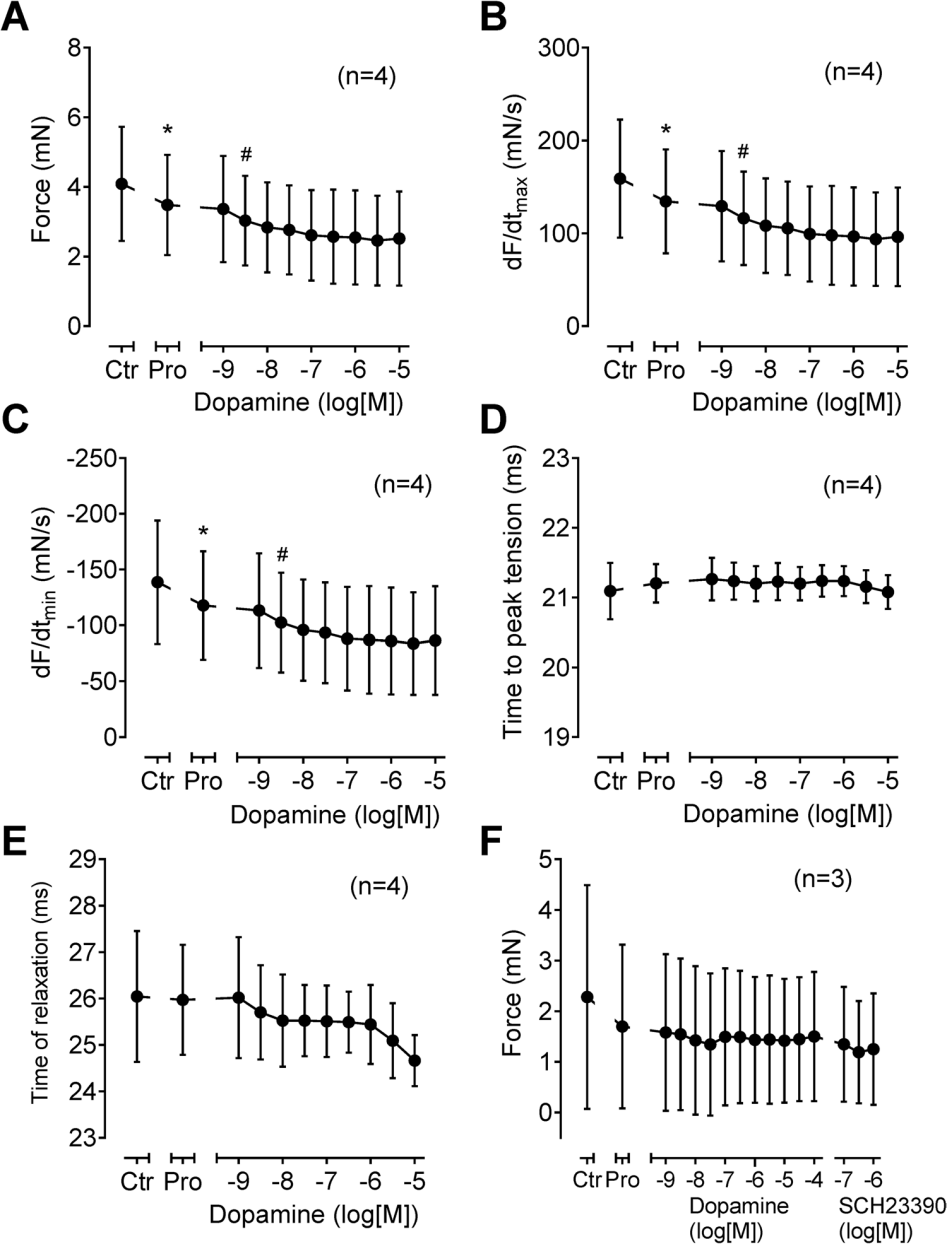
Contractile effects of dopamine in left atrial preparations from WT. Several experiments on isolated electrically stimulated left atrial preparations from WT such as in Fig. 4 are summarized here. Ordinates in (**A**) and (**F**) give force of contraction in millinewton (mN). Ordinates in (**B**) and (**C**) give rate of contraction and rate of relaxation in millinewton per second (mN/s). Ordinates in (**D**) and (**E**) give time to peak tension and time of relaxation in milliseconds (ms). Abscissae in this figure indicate first application of 10 µM propranolol (Pro) and subsequent concentrations of dopamine in negative decadic molar concentrations. * and # indicate significant differences versus control (Ctr; pre-drug value) or propranolol, respectively. “*n*” indicates number of experiments

In initial experiments, we noted with dopamine in the absence of propranolol a positive inotropic effect, presumably via β-adrenoceptors consistent with the literature (data not shown). In the presence of 10 µM propranolol (used to block adrenergic effects of dopamine), dopamine failed to increase force of contraction in WT. This is depicted in an original recording in a left atrial preparation in Fig. 4A. The results in WT are summarized in Fig. 5A for force of contraction, in Fig. 5B for the rate of tension development, and in Fig. 5C for the rate of relaxation: propranolol per se, as expected, reduced these parameters, but they were not augmented by dopamine. In the same samples, dopamine minimally shortened time to peak tension and time of relaxation (Fig. 5 D and E). Correspondingly, the D_1_ receptor antagonist SCH23390 had no effect in WT preparations (Fig. 5F).

**Fig. 6:**
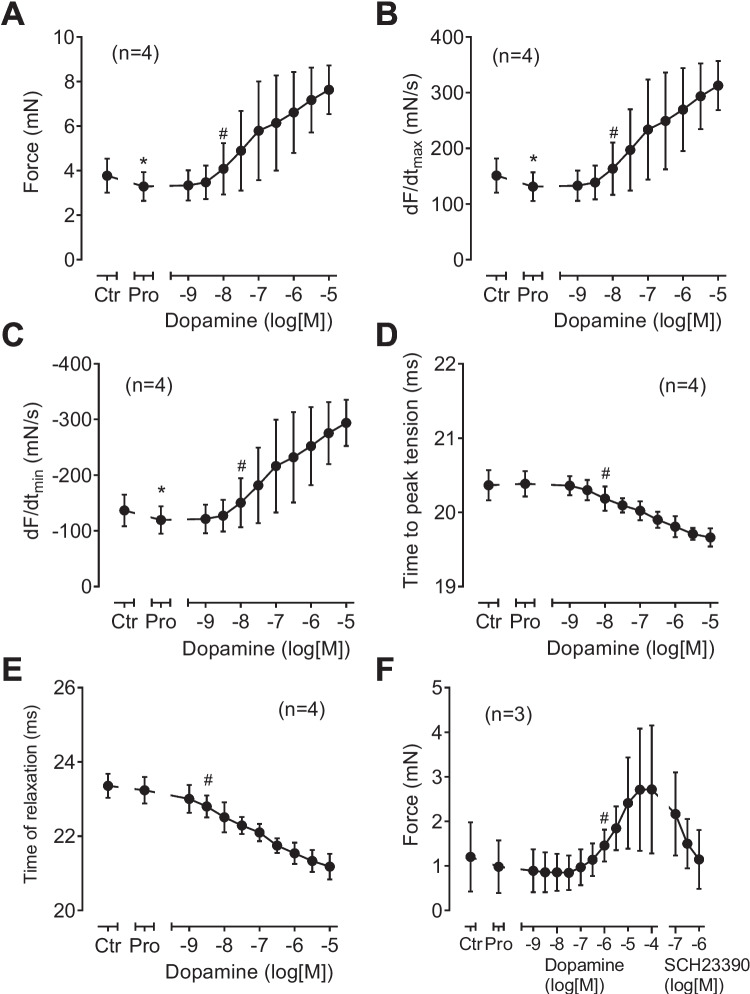
Contractile effects of dopamine in left atrial preparations from D_1_-TG. Several experiments on isolated electrically stimulated left atrial preparations from D_1_-TG such as in Figure 4 are summarized here. Ordinates in (**A**) and (**F**) give force of contraction in millinewton (mN). Ordinates in (**B**) and (**C**) give rate of contraction and rate of relaxation in millinewton per second (mN/s). Ordinates in (**D**) and (**E**) give time to peak tension and time of relaxation in milliseconds (ms). Abscissae in this figure indicate first application of 10 µM propranolol (Pro) and subsequent concentrations of dopamine in negative decadic molar concentrations. * and # indicate significant differences versus control (Ctr; pre-drug value) or propranolol, respectively. “*n*” indicates number of experiments

However, in D_1_-TG, dopamine was effective to increase force of contraction. This is plotted as an original recording in Fig. 4B. The results are summarized in Fig. 6A for force of contraction, in Fig. 6B for the rate of tension development, and in Fig. 6C for the rate of relaxation: propranolol, as expected reduced these parameters, but they were augmented by dopamine. In the same samples, dopamine substantially shortened time to peak tension and time of relaxation (Fig. 6 D and E). Moreover, the positive inotropic effects of dopamine were antagonized by SCH23390 (Fig. 6F). Additionally, dopamine was equi- equi-efficacious as isoprenaline to increase force of contraction in D_1_-TG. Force of contraction in D_1_-TG was increased to a maximum of 109 ± 35% (*n* = 3) by dopamine, if the maximum effect of isoprenaline was normalized to 100% (There was no dopamine effect in WT).

**Fig. 7:**
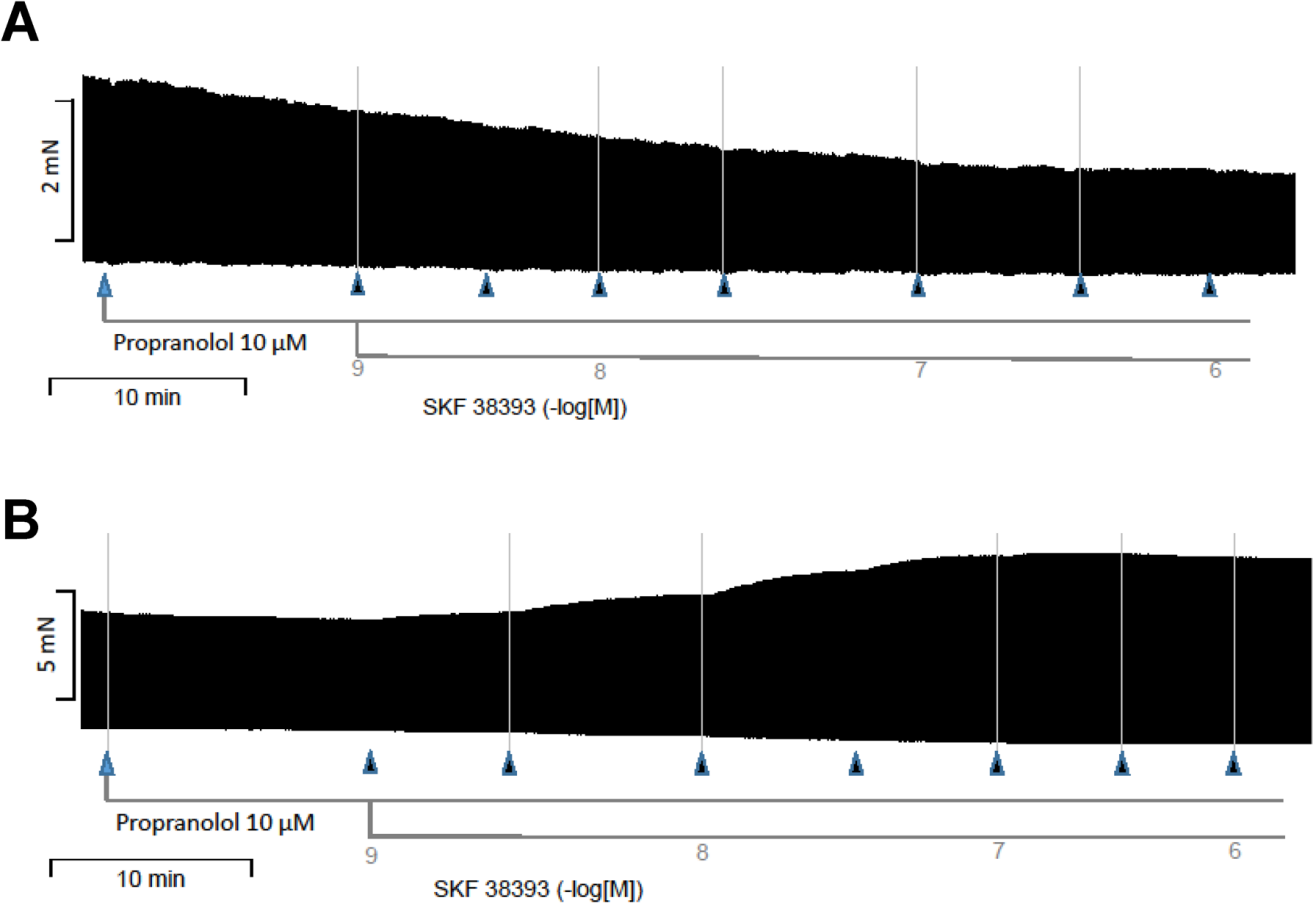
Original recordings: effect of SKF 38393 on left atrial preparations in WT (**A**) and D1-TG (**B**). Isolated electrically stimulated left atrial preparations from WT (**A**) and D_1_-TG (**B**) were studied. Ordinates give force of contraction in millinewton (mN). Horizontal bar indicates time in minutes (min). First propranolol alone was given (10 µM). After 10 min of pre-treatment, SKF 38393 was cumulatively applied. Time points of addition of SKF 38393 are indicated with arrows pointed upwards. Concentrations of SKF 38393 are indicated as negative decadic logarithms. SKF 38393 increases force of contraction only in preparations from D_1_-TG

**Fig. 8:**
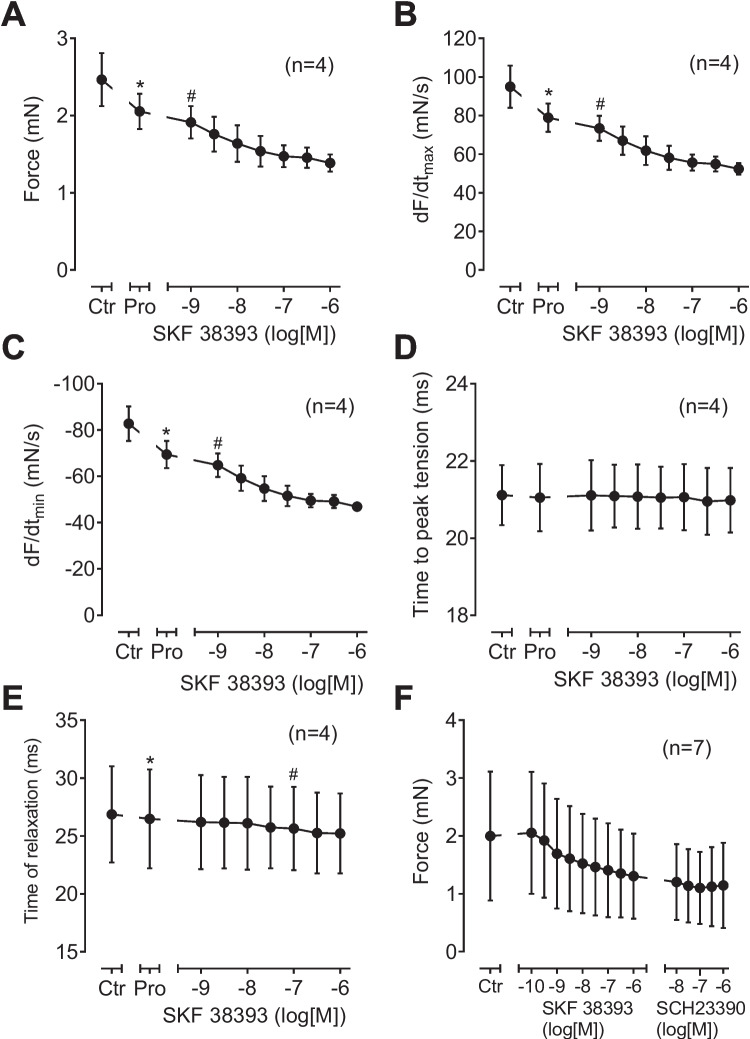
Contractile effects of SKF 38393 in left atrial preparations from WT. Several experiments such as in Fig. 7A and, in addition, with a final concentration-response-curve with the D_1_ receptor antagonist SCH23390 are summarized here. Isolated electrically stimulated left atrial preparations from WT were studied. Ordinates in (**A**) and (**F**) give force of contraction in millinewton (mN). Ordinates in (**B**) and (**C**) give rate of contraction and rate of relaxation in millinewton per second (mN/s). Ordinates in (**D**) and (**E**) give time to peak tension and time of relaxation in milliseconds (ms). Abscissae in this figure indicate first application of 10 µM propranolol (Pro) and subsequent concentrations of SKF 38393 in negative decadic molar concentrations. * and # indicate significant differences versus control (Ctr; pre-drug value) or propranolol, respectively. “*n*” indicates number of experiments

SKF 38393 (a benzazepine derivative, that contains like dopamine a dihydroxy-benzene moiety and an amino group linked by two carbon atoms to this benzene ring system), and a known agonist at D_1_- and D_5_-receptors (Neumann et al. 2023a), did not increase force of contraction in WT. This is depicted as an original recording in Fig. 7A. The results for WT are summarized in Fig. 8A for force of contraction, in Fig. 8B for the rate of tension development, and in Fig. 8C for the rate of relaxation: propranolol, as expected reduced these parameters. In these same samples, SKF 38393 did not shorten time to peak tension and slightly reduced time of relaxation (Fig. 8 D and E). As shown above for dopamine, SCH23390 again had no effect in WT preparations (Fig. 8F).

**Fig. 9:**
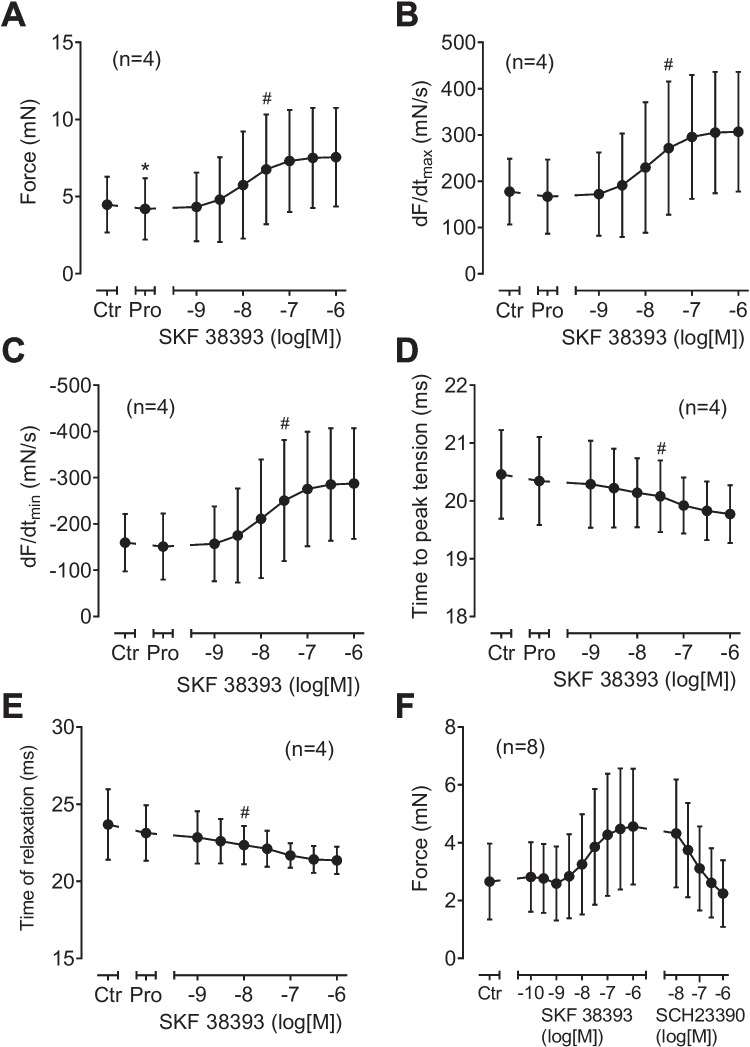
Contractile effects of SKF 38393 in left atrial preparations from D_1_-TG. Several experiments such as in Fig. 7B and, in addition, with a final concentration-response-curve with the D_1_ receptor antagonist SCH23390 are summarized here. Isolated electrically stimulated left atrial preparations from D_1_-TG were studied. Ordinates in (**A**) and (**F**) give force of contraction in millinewton (mN). Ordinates in (**B**) and (**C**) give rate of contraction and rate of relaxation in millinewton per second (mN/s). Ordinates in (**D**) and (**E**) give time to peak tension and time of relaxation in milliseconds (ms). Abscissae in Fig. 9 indicate first application of 10 µM propranolol (Pro) and subsequent concentrations of SKF 38393 in negative decadic molar concentrations. * and # indicate significant differences versus control (Ctr; pre-drug value) or propranolol, respectively. “*n*” indicates number of experiments

SKF 38393 increased force of contraction in D_1_-TG in a concentration- and time-dependent manner (Fig. 7B). This is depicted as an original recording in Fig. 7B. The results are summarized for left atrial preparations from D_1_-TG in Fig. 9A for force of contraction, in Fig. 9B for the rate of tension development, and in Fig. 8C for the rate of relaxation: propranolol, as expected reduced these parameters (e.g., Fig. 9A), but they were augmented by SKF 38393. In the same samples, SKF 38393 substantially shortened time to peak tension and time of relaxation (Fig. 9 D and E). The positive inotropic effects of SKF 38393 were antagonized by SCH23390 (Fig. 9F).

### Beating rate in right atrium of D_1_-TG

**Fig. 10:**
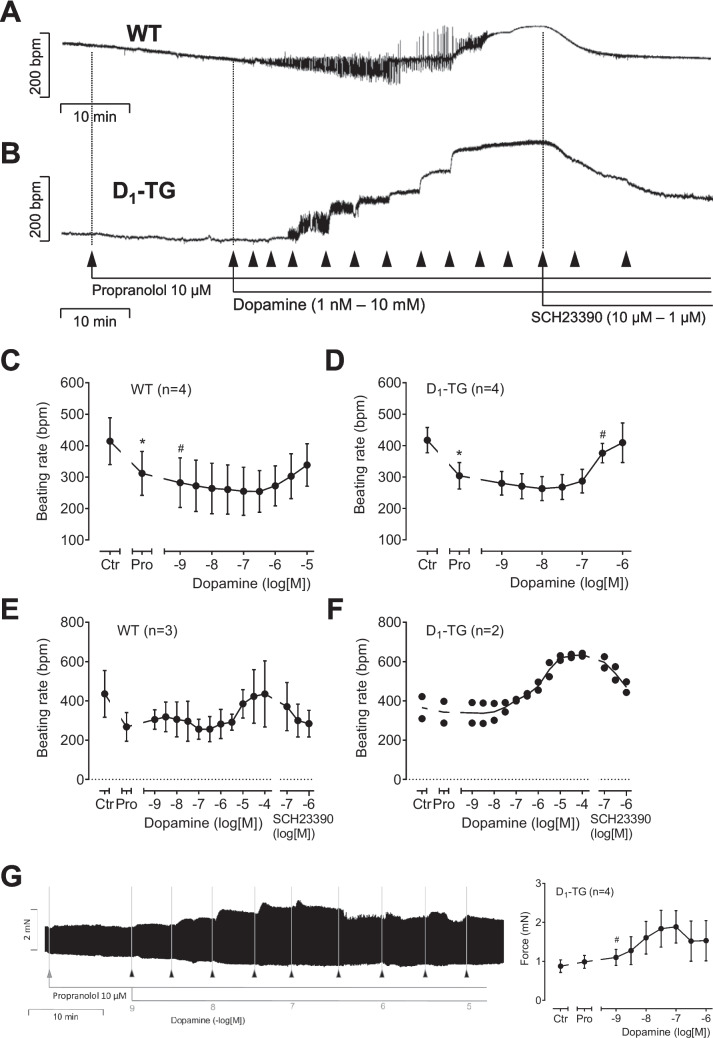
Original recordings of the effects of dopamine in right atrial preparations from WT (**A**) and D_1_-TG (**B**, **C**). Isolated spontaneously beating right atrial preparations (RA) from WT (**A**) and D_1_-TG (**B**, **G**) were studied. Ordinates in (**A**) to (**F**) give beating rates in beats per minute (bpm). Ordinates in (**G**) give force of contraction in millinewton (mN). Horizontal bars indicate time in minutes (min) in (**A**), (**B**), and (**G**). First, propranolol alone was given (10 µM). After 10 min of pre-treatment, in addition dopamine was cumulatively applied. Finally, the D_1_ receptor antagonist SCH23390 was cumulatively applied. Time points of addition of dopamine or SCH23390 are indicated with arrows pointed upwards. Concentrations of dopamine in Fig. 10G are indicated as negative decadic logarithms. Dopamine increases beating rate and force in right atrial preparations from D_1_-TG. Abscissae in thid figure indicate first application of 10 µM propranolol (Pro) and subsequent concentrations of dopamine in negative decadic molar concentrations. * and # indicate significant differences versus control (Ctr; pre-drug value) or propranolol, respectively. “*n*” indicates number of experiments

When one looks closely at the effect of increasing dopamine concentrations on right atrial force of contraction and beating rate, one detects in right atrial preparations from both D_1_-TG (Fig. 10B) and WT (Fig. 10A) a certain positive chronotropic inotropic effect. However, as would be expected, the chronotropic effect occurs at lower concentrations of dopamine and is more effective in D_1_-TG (Fig. 10B, D) compared to WT (Fig. 10A, C). Moreover, these positive chronotropic effects are attenuated by increasing concentrations of SCH23390 and thus are D_1_-mediated (Fig. 10E, F). The positive chronotropic effects of dopamine in right atrial preparations were accompanied by an increase in force of contraction in these right atrial preparations. This was unexpected because in mouse right atrial preparations, an increase in the beating rate per se reduces force of contraction (negative staircase). Hence, this negative staircase effect is superceded by a direct positive inotropic effect of dopamine (Fig. 10G). Interestingly, in contrast to force of contraction, dopamine was equi-efficacious as isoprenaline to increase the beating rate in WT as well as in D_1_-TG. The beating rate was increased by dopamine to a maximum of 102 ± 10% (*n* = 3) in D_1_-TG and to a maximum of 105 ± 42% (*n* = 3) in WT if the maximum effect of isoprenaline was normalized to 100%.

**Fig. 11:**
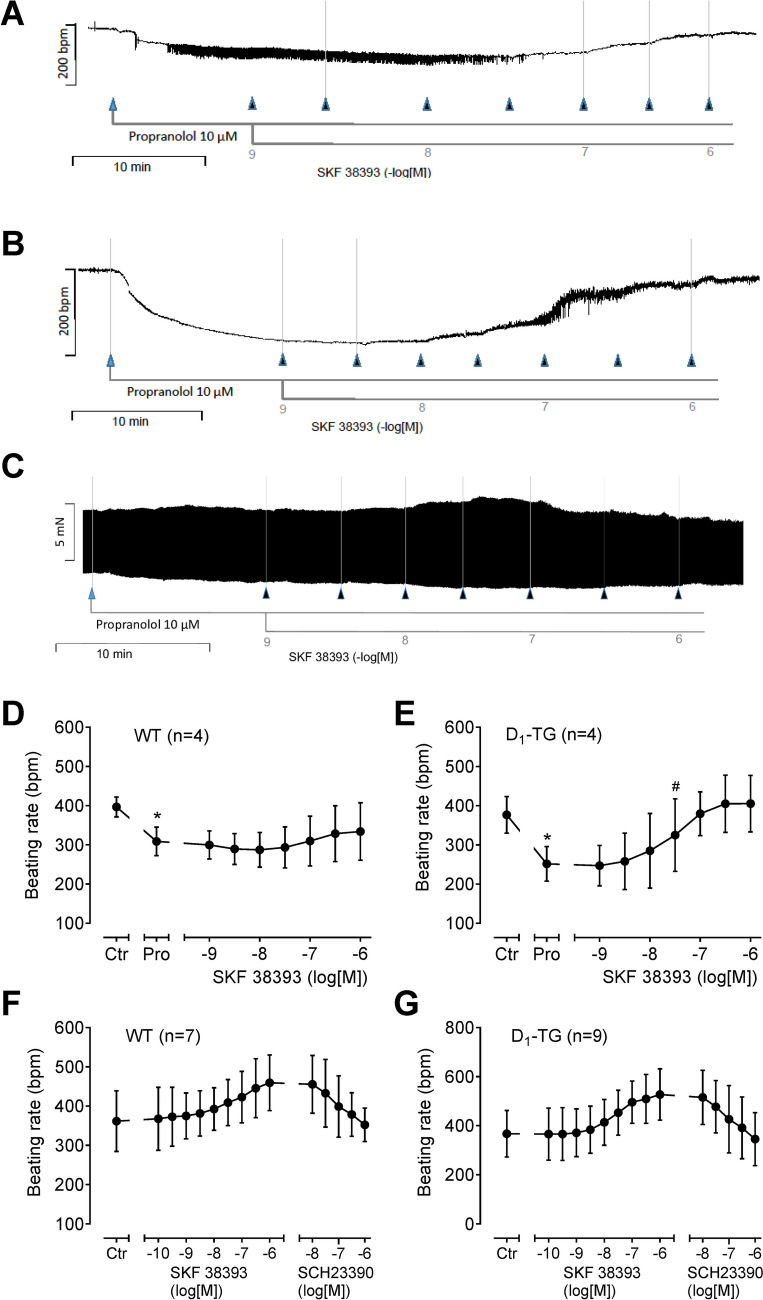
Original recordings of SKF 38393 in right atrial preparations from WT (**A**) and D_1_-TG (**B**, **C**). Isolated spontaneously beating right atrial preparations (RA) from WT (**A**) and D_1_-TG (**B**, **C**) were studied. Ordinates in (**A**), (**B**), and Fig. 10D give beating rates in beats per minute (bpm). Ordinate in (**C**) gives force of contraction in millinewton (mN). Horizontal bars indicate time in minutes (min) in (**A**), (**B**), and (**C**). First propranolol alone was given (10 µM). After 10 min of pre-treatment, in addition SKF 38393 was cumulatively applied. Time points of addition of SKF 38393 are indicated with arrows pointed upwards in (**A**), (**B**), and (**C**). Concentrations of SKF 38393 are indicated as negative decadic logarithms. SKF 38393 increases beating rate and force in right atrial preparations from D_1_-TG. In (**F**) and (**G**), the D_1_ receptor antagonist SCH23390 was cumulatively applied after SKF 38393. Abscissae in this figure indicate first application of 10 µM propranolol (Pro) and subsequent concentrations of SKF 38393 in negative decadic molar concentrations. * and # indicate significant differences versus control (Ctr; pre-drug value) or propranolol, respectively. “*n*” indicates number of experiments

The same as with dopamine was noted with SKF 38393 in right atrial preparations. As seen, SKF 38393 increased the beating rate in right atrial preparations from WT (Fig. 11A). However, SKF 38393 was more potent and effective to raise beating rate in right atrial preparations from D_1_-TG. Again, these positive chronotropic effects are attenuated by increasing concentrations of SCH 23390 and thus are D_1_-mediated (Fig. 11E, F). This indicates that there is an endogenous D_1_-dopamine receptor in WT that can be stimulated under our experimental conditions, but this is a marginal effect compared to the stimulation of D_1_-dopamine-receptors in D_1_-TG.

### Langendorff perfusion

Finally, the question arose whether the overexpression of the D_1_-dopamine receptor also altered ventricular performance. This is clinically relevant because the cardiac ejection fraction is mainly determined by the ventricles. We measured force therefore in the apex of the left ventricle. We notice that in the presence of 0.4 µM propranolol, 1 µM dopamine increased after 10 min the force of contraction and the rate of relaxation in D_1_-TG but not WT (Table 1). Thus, we have functionally overexpressed the D_1_-dopamine receptor not only in the atria but also in the left ventricle (Table 1). As concerns the beating rate, the basal beating rate was higher (but not significantly) in D_1_-TG compared to WT (Table 1). From these values, heart rate was increased significantly in WT but not D_1_-TG. The increase in heart rate in WT is in line with our findings in right atrial preparations (cf. Fig. 10A).

**Table 1:** Maximum effect of 1 µM dopamine in the presence of 0.4 µM propranolol on force of contraction in millinewton (mN), on the rate of tension relaxation in mN/seconds (mN/s) and on the beating rate in beats per minute (bpm) in isolated perfused hearts from D_1_-TG and WT. # indicate *p* < 0.05 versus pre-drug (before dopamine) value. *N* number of animals

	WT	D_1_-TG
N	5	4
Basal force (mN)	12.1 ± 7.4	8.5 ± 3.3
Force after dopamine (mN)	10.3 ± 7.2	13.8 ± 12.7#
Basal rate of relaxation (mN/s)	− 221 ± 145	− 153 ± 54
Rate of relaxation after dopamine (mN/s)	− 177 ± 123	− 281 ± 107#
Basal beating rate (bpm)	242 ± 49	284 ± 33
Beating rate after dopamine (bpm)	323 ± 69#	342 ± 59

## Discussion

### Main new findings

The main new finding is the establishment of a mouse model to study cardiac effects of D_1_-dopamine receptor. Using this model, we could show that stimulation of the overexpressed human D_1_-dopamine receptor in the mammalian heart can in principle lead to a positive chronotropic and positive inotropic effect.

We confirmed the generation of this D_1_-TG by detecting the DNA in the tail biopsies in D_1_-TG but not in WT. Moreover, in the heart, we could detect huge mRNA for the human D_1_-dopamine receptor in the heart of D_1_-TG but hardly any signal in WT, as expected. On Western blots, we noted no change in the expression of important cardiac regulatory proteins like PLB, TnI and SERCA between D_1_-TG and WT.

Moreover, we noted a positive inotropic effect in left atrial preparations from D_1_-TG with the pleiotropic agonist dopamine. In this context, one could argue that dopamine might activate all dopamine-receptors. Therefore, we also tested the more selective dopamine receptor agonist SKF 38393. This compound does not stimulate D_2_-, D_3_-, D_4_-dopamine receptors. SKF 38393, however, does not only stimulate D_1_-dopamine receptors but also D_5_-dopamine receptors (review: Neumann et al. 2023b). Moreover, we could the positive inotropic effect of dopamine and SKF 38393 could be antagonized by the antagonist SCH 23390. Here, a similar uncertainty is clear. SCH 23390 does not only act as an antagonist at D_1_- but also D_5_-dopamine receptors (Neumann et al. 2023b).

However, our data cumulatively argue for a D_1_-dopamine receptor-mediated effect in D_1_-TG. The evidence is based on the fact that we overexpressed the D_1_-dopamine receptor and this overexpressed was supported by the data of the RT-PCR, the in-situ hybridization experiments, and the functional contractile studies.

Interestingly, dopamine led to stimulation of the beating rate in D_1_-TG with a similar potency as it increased the force of contraction in D_1_-TG. Interestingly from a physiological point of view, even in WT we noted an increase in the beating rate. This increase in WT in the beating rate by dopamine occurred at higher concentrations that in D_1_-TG. This is consistent with our data from RT-PCR, that mouse heart contains D_1_-dopamine receptor. However, others before published that mouse heart does not only express as mRNA D_1_-dopamine receptor but also D_5_-dopamine receptor. Moreover, it is plausible that dopamine was more potent in D_1_-TG than in WT because the mRNA data might be extrapolated to protein data and we would argue that when more D_1_-dopamine receptor are present in the right atrium of D_1_-TG versus WT then less dopamine is required to exert a similar chronotropic effect. As far as we know, a D_1_-dopamine receptor-mediated positive chronotropic effect in mouse atrium has not yet been reported, probably because others overlooked this small effect.

Hence, it needs to be elucidated whether the increase in beating rate is really mediated by D_1_-dopamine receptor or D_5_-dopamine receptor. This could be answered by performing these contractions experiments anew in right atrial preparations from D_1_-KO and separately in D_5_-KO. Both D_1_-KO and D_5_-KO have been described and exist, but these studies were beyond the scope of the present study.

Finally, we wanted to contribute to a better understanding of the signal transduction on the D_1_-dopamine receptor in the heart. If we hypothesize that D_1_-dopamine receptors couple in the cardiomyocytes exactly as β-adrenergic receptors (Fig. 1), one would expect an increase in the phosphorylation state of TnI and this is just what we have measured. These increased phosphorylations of TNI could at least in part explain the relaxant effects of dopamine in the heart via D_1_-dopamine receptor. TnI is only expressed in cardiomyocytes. The fact that we detect an increased phosphorylation of TnI with an antibody that is specific of the cAMP-dependent phosphorylation of TnI on amino acids 22/23 argues that our data are consistent with the view that D1-dopamine receptors signal via cAMP under our experimental conditions.

The fact that dopamine increased (in the presence of propranolol) the force in the left ventricle of the mouse (Langendorff) would argue that the D_1_-dopamine receptor might potentially be relevant in human heart failure usually characterized by systolic left ventricular failure.

### Clinical relevance

As mentioned above, D_1_-dopamine receptors are present in the human heart and their expression, perhaps as a compensatory mechanism, increases in heart failure but also in ventricular arrhythmias (Yamaguchi et al. 2020). The question arises whether dopamine can increase force of contraction via D_1_-dopamine receptors in the human heart. D_1_-dopamine receptors have been repeatedly identified in the human heart using Western blotting, immunohistochemistry, or mRNA detection. This has been done in whole heart homogenates and heart slices (Amenta et al. 1993, Cavallotti et al. 2010, Tonnarini et al. 2011). Moreover, in human-induced-pluripotent stem cell-derived cardiomyocytes one detected D_1_-dopamine receptors in Western blots as protein and on mRNA level using PCR (Huang et al. 2022). They also found prolongation of action potentials and arrhythmias in these cells upon treatment with fenoldopam, a D_1_-dopamine receptor agonist, and to SKF38393 (Huang et al. 2022). This could mean that D_1_-dopamine receptor is functional in the human heart. However, they could not ascribe functional effects solely to D_1_-dopamine receptors because no selective D_1_-dopamine receptor agonist or D_1_-dopamine receptor antagonist are currently known. Moreover, they did not measure force of contraction or cell movement in their interesting model (Huang et al. 2022). Hence, in this regard, our D_1_-TG model is especially valuable, as we can be confident that in this model, our increases in force of contraction are really D_1_-dopamine receptor mediated: while we also use the dual D_1_/D_5_ agonist SKF38393, we have only overexpressed D_1_-dopamine receptors and would thus argue that we measured bona fide responses via D_1_-dopamine receptors.

Moreover, as concerns function in the human heart, others detected in human papillary muscles that haloperidol could shift the concentration response curve for the positive inotropic effect of dopamine to the right (Brown et al. 1985). Hence, there are published data that the D_1_-dopamine receptor might couple to force of contraction in the human heart. However, haloperidol blocks not only D_1_-dopamine receptors but also D_2_-dopamine receptors (review: Neumann et al. 2023). Thus, more specialized contractile studies are clearly needed as soon as new D_1_-dopamine receptor agonists or D_1_-dopamine receptor antagonists become available.

As concerns a putative physiological role of dopamine in the human heart, it seems of interest to mention that dopamine is present in the human heart (Regitz et al. 1990). Dopamine could perhaps be even formed in the human heart (Czibik et al. 2021). Hence, if dopamine was released from human cardiomyocytes, it might stimulate D_1_-dopamine receptors in an autocrine way in the human heart. But this is also hypothetical and needs experimental confirmation which we could provide in part with our D_1_-TG model.

Hence, our model could perhaps be used to study the clinical role of D_1_-dopamine receptor. Moreover, our model might assess the action of clinically used drugs on D_1_-dopamine receptors in a functional way in the heart. If it is true that D_1_-dopamine receptor stimulation leads to arrhythmias in patients, Parkinson drugs that potently stimulate D_1_-dopamine receptor might be detrimental. Our model offers moreover the option to test how selective an agonist (or an antagonist) is functionally for D_1_-dopamine receptor. A last application of D_1_-TG may lie in psychiatry. Many neuroleptics are potent antagonists at D_1_-dopamine receptor and we are planning contraction studies with such drugs in D_1_-TG to find out whether this would reduce the potency of dopamine to exert contractile effects via D_1_-dopamine receptor.

## Limitations of the study

We noted a positive chronotropic effect of dopamine in the presence of propranolol in Langendorff preparations from WT. This might mean stimulation of endogenous D_1_ receptors in WT (see Fig. 10A). It is well known that the density of, for instance, β-adrenoceptors is higher in sinus node than in working myocardium. Hypothetically, this might be true for the D_1_-dopamine receptor and therefore we detected an increase in beating rate in WT. However, biochemical data on the expression of D_1_-dopamine receptors in the sinus node of WT and D_1_-TG are currently lacking. In the atrial preparations of D_1_-TG, we noted a potent positive chronotropic effect to dopamine in the presence of propranolol (Fig. 10B). However, this was not the case in the Langendorff preparation of D_1_-TG. There was only a tendency. This is possibly a methodological problem: the experimental conditions (gassing, temperature, isometric set-up) are much better controlled in the mouse atrial preparation that spontaneously contracts in the organ bath than in the isolated heart in the Langendorff system. In our perfused heart set-up, the heart is fixed at the aorta and contracts in the ambient air. Hence, with a more sophisticated Langendorff system (the heart might be immersed in an organ bath), we expect to be able to obtain a positive chronotropic effect in this transgenic model with dopamine (in the presence of propranolol).

We have no drugs at our disposal that selectively stimulate or block D_1_-dopamine receptor and not also D_5_-dopamine receptor. Such drugs are awaited with interest. Moreover, under normal conditions in vivo, dopamine would be expected to stimulate a mix at least of dopamine receptors, β_1_- and β_2_-receptors and possibly α-adrenoceptors in the human heart. Therefore, our data cannot prove that the D_1_-dopamine receptor in the human cardiomyocytes exerts a positive inotropic effect or in the human sinus node cell induces a positive chronotropic effect.

Nevertheless, to answer our initial hypothesis, we detect a major positive inotropic and chronotropic effect of dopamine mediated by human D_1_-dopamine receptors in our model system.

## Data Availability

The data of this study are available from the corresponding author upon reasonable request.
